# Supplementary material for: Performance Enhancement of All-Inorganic Carbon-Based CsPbIBr2 Perovskite Solar Cells Using a Moth-Eye Anti-Reflector
Source: Nanomaterials (Basel). 2021 Oct 15;11(10):2726. doi: 10.3390/nano11102726 (PMC8538339; doi:10.3390/nano11102726)
Supplement: Supplementary file 1 [file nanomaterials-11-02726-s001.zip › nanomaterials-1390620-supplementary.pdf]

Supplementary Materials

Performance Enhancement of All-Inorganic Carbon-Based $CsPbIBr_2$ Perovskite Solar Cells Using a Moth-Eye Anti-Reflector

Wensheng Lan [1], Dazheng Chen [1,*], Qirui Guo [1], Baichuan Tian [1], Xiaoping Xie [2], Yibing He [1], Wenming Chai [1], Gang Liu [2], Peng Dong [2], He Xi [1], Weidong Zhu [1] and Chunfu Zhang [1,*]

[1] State Key Discipline Laboratory of Wide Band Gap Semiconductor Technology, Xidian University, Xi'an 710071, China; lws85626637@163.com (W.L.); guoqirui1014@163.com (Q.G.); tbcxd0515@yeah.net (B.T.); heyibing0507@163.com (Y.H.); 15009205592@163.com (W.C.); hxi@xidian.edu.cn (H.X.); wdzhu@xidian.edu.cn (W.Z.)
[2] Qinghai Huanghe Hydropower Development CO., LTD., Xining 810008, China; xiexiaoping@spic.com.cn (X.X.); liugang@spic.com.cn (G.L.); dongpeng@spic.com.cn (P.D.)
* Correspondence: dzchen@xidian.edu.cn (D.C.); cfzhang@xidian.edu.cn (C.Z.)

Part 1 Supplementary Figures and Table

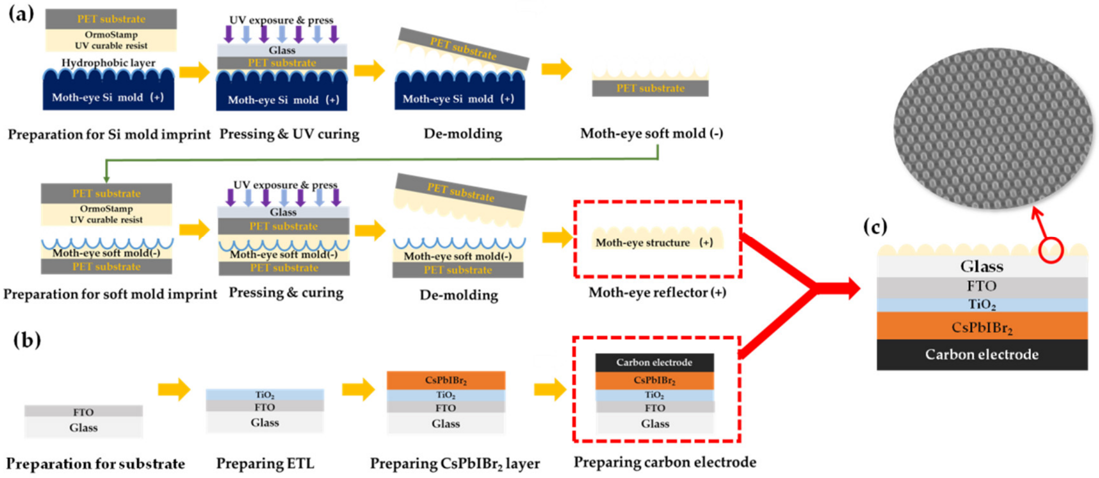

Figure S1: Schematic diagram of preparation process for **(a)** moth-eye anti-reflector and **(b)** all-inorganic $CsPbIBr_2$ PSCs. **(c)** Fabricated all-inorganic $CsPbIBr_2$ PSCs with a moth-eye anti-reflector.

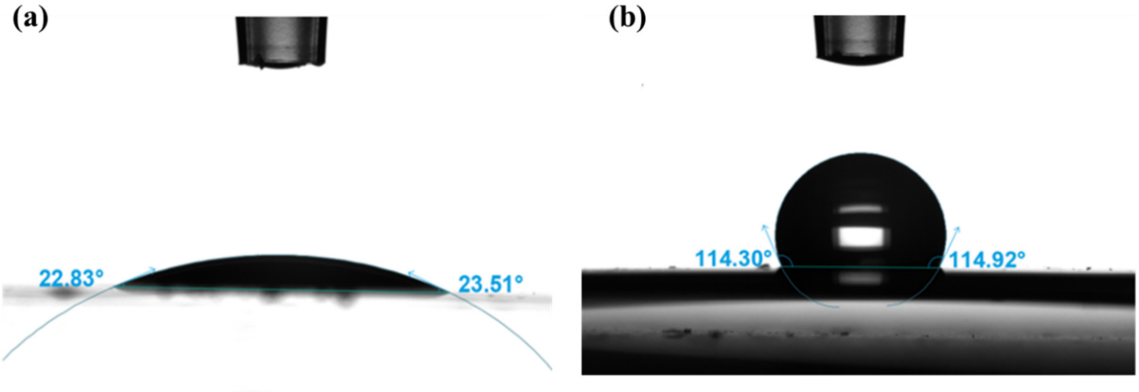

Figure S2: Water contact angle diagram of **(a)** glass and **(b)** moth-eye nanostructure on glass substrate.

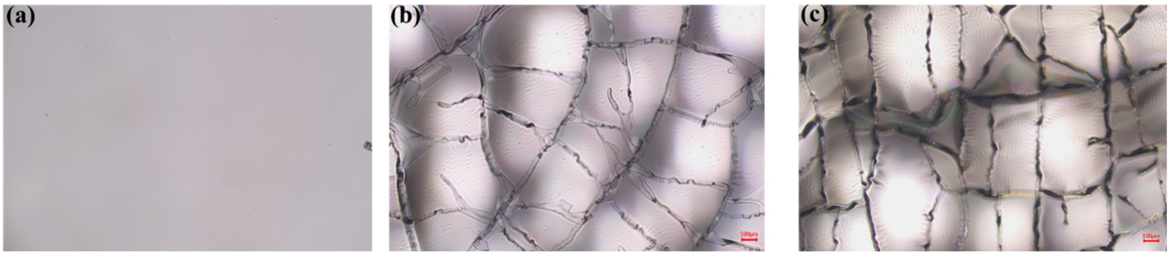

Figure S3: Crack of OrmoStamp film with moth-eye structure at (**a**) 220°C、(**b**)230°C and (**c**)240°C.

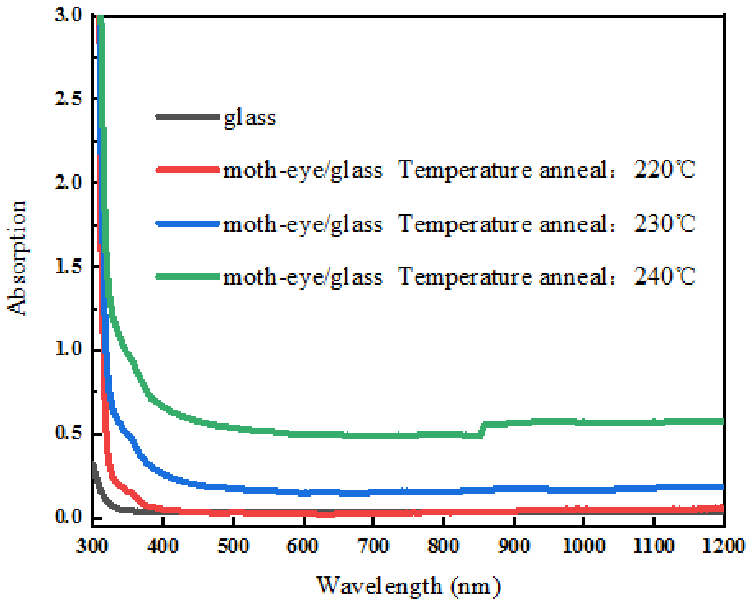

Figure S4: Parasitic absorption of OrmoStamp film with moth-eye structure at (**a**) 220°C、(**b**)230°C and (**c**)240°C.

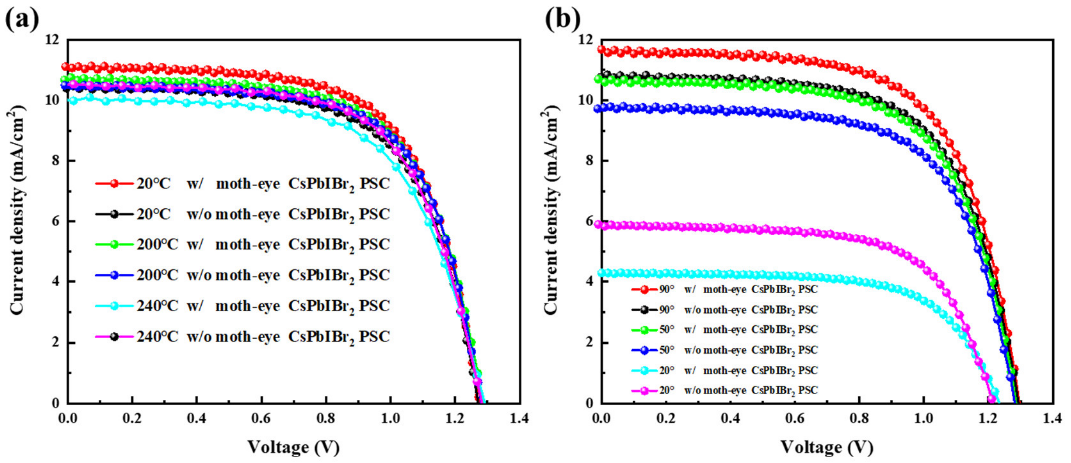

Figure S5: J-V curves with and without moth-eye structure devices at different (**a**) temperatures and (**b**) incident angles.

Table S1: Key methods, J_{SC}s and PCEs of all the reported $CsPbIBr_2$-based solar cells in the literature and in this work.

Cell configuration	Key methods	V_{OC} [V]	J_{SC} [mA/cm²]	FF [%]	PCE [%]	Ref
FTO/c-TiO_2/ $CsPbIBr_2$/Carbon	**Moth-eye structure**	1.28	**11.91**	66	10.10	**This work**
FTO/ c-TiO_2/ $CsPbIBr_2$/Carbon	aged precursor	1.14	9.11	63	6.55	[24]
FTO/ c-TiO_2/ $CsPbIBr_2$/Carbon	Intermolecular exchange	1.25	10.66	69	9.16	[8]
ITO/Passivated SnO_2/ $CsPbIBr_2$/Carbon	$SnCl_2$ solution passivation	1.23	8.5	67	7.00	[54]
FTO/ c-TiO_2/ $CsPbIBr_2$/carbon	Light-processing	1.28	11.17	60	8.60	[26]
ITO/SnO_2/ $CsPbIBr_2$/carbon	One step	1.07	7.55	58	4.71	[54]
FTO/c-TiO_2/ $CsPbIBr_2$/carbon	One step	1.21	11.37	63	8.68	[18]
FTO/c-TiO_2/CsBr/ $CsPbIBr_2$/Carbon	CsBr modified TiO_2 layer	1.26	11.8	72	10.71	[18]
FTO/ c-TiO_2/ $CsPbIBr_2$/Carbon	Flux-mediated growth	1.32	11.68	70	10.82	[25]
FTO/ c-TiO_2/ $CsPbIBr_2$/PMMA/Carbon	PMMA modification	1.30	11.36	62	9.21	[55]

Part 2 FDTD Simulation Method and Result

Method. The finite-difference time-domain (FDTD) tool, which is based on solving a set of Maxwell's curl equations, as shown by the equation 1, to simulate the propagation of incident light as well as the interactions between electromagnetic waves and the structure. The detail introduction of FDTD method can be found in references [36,48-49,59].

$$\begin{cases} \nabla \times \mathbf{H} = \frac{\partial \mathbf{D}}{\partial \mathrm{t}} \\ \nabla \times \mathbf{E} = -\frac{\partial \mathbf{B}}{\partial \mathrm{t}} \\ \mathbf{B} = \mu \boldsymbol{H} \\ \boldsymbol{D} = \varepsilon \boldsymbol{E} \end{cases} \quad (1)$$

Where, **H**, **D**, **E**, **B**, μ and ε represent the magnetic field, the electric displacement, the electric field, the magnetic flux density, complex permeability and complex permittivity.

Results. To further exploit the potential of moth-eye nanostructure, we established the optical model and performed the simulation optimization by the finite-difference time-domain (FDTD) tool. It is known that the size, period, and shape are key parameters to adjust the optical performance of nanostructures. In Figure S6a-d, for the moth-eye anti-reflector with a single parabolic surface, the optimized parameters include a ratio of depth to width of 2.5, a line width of 300 nm, and a period of 450 nm. The corresponding average reflectance dropped to 0.64 % within the wavelength range from 350 nm to 800 nm, and thus could be used to further improve the performance of $CsPbIBr_2$ PSCs. On the other hand, considering the requirements of wide-range anti-reflection in double and triple-junction tandem solar cells, the reflectance from 200 nm to 1600 nm has been calculated for single and composite moth-eye nanostructures. It can be noted from Figure 7e-f that, although significantly lower than glass, the reflectance of single moth-eye nanostructure was markedly increased at longer wavelength. While for the composite moth-eye nanostructure the average reflectance could be further reduced to only 0.74% in the wide wavelength range from 200 nm to 1600 nm, which is instructive to the anti-reflector design and performance enhancement for the single and multi-junction solar cells.

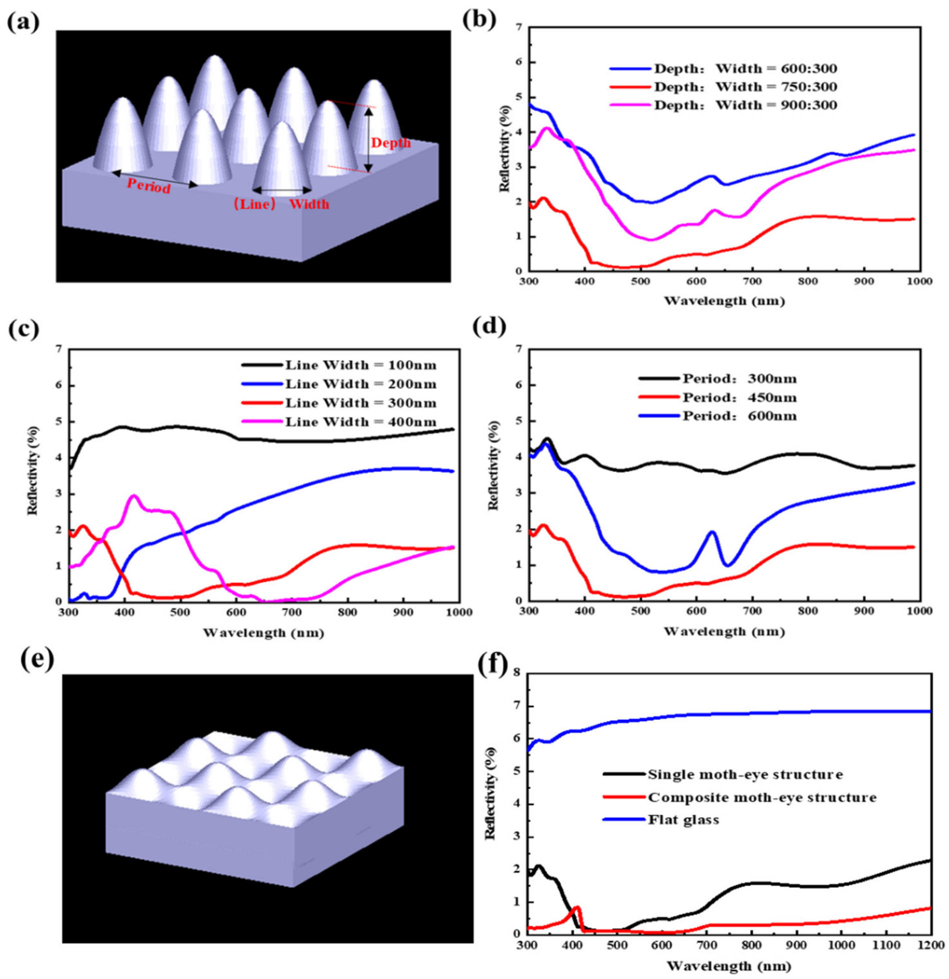

Figure S6. Schematic graph of a (a) single and (e) composite moth-eye nanostructure simulated by FDTD. Reflectivity at different (b)ratios of depth to width, (c) line width and (d) period; (f) Reflectivity curves of flat glass, single and composite moth-eye structures.
